# Factors Influencing Visual Improvement after Phacoemulsification Surgery among Malaysian Cataract Patients

**DOI:** 10.3390/ijerph191811485

**Published:** 2022-09-13

**Authors:** Nadiah Sa’at, Anis Kausar Ghazali, Najib Majdi Yaacob, Mohamad Aziz Salowi

**Affiliations:** 1 Biostatistics and Research Methodology Unit, School of Medical Sciences, Health Campus, Universiti Sains Malaysia, USM, Kubang Kerian 16150, Malaysia; 2 Hospital Selayang, Batu Caves 68100, Malaysia

**Keywords:** affecting factors, improvement, multivariable analysis, visual acuity

## Abstract

Blindness and visual impairment are part of the global burden of eye disease, with cataract being one of the leading causes of blindness. This study aimed to determine the factors affecting visual acuity (VA) improvement among cataract patients after phacoemulsification surgery in Malaysia. Cataract patients aged over 18 who underwent phacoemulsification surgery between January 2014 and December 2018 were included in this retrospective cohort study. Patients’ sociodemographic, comorbidities, surgical, and related complication factors were extracted from the National Eye Database. The outcome was measured by the difference in visual acuity before and after the operation and was categorized as “improved”, “no change”, and “worse”. A total of 180,776 patients were included in the final analysis. Multinomial logistic regression analysis showed “no changes in VA” was significantly higher in patients aged less than 40 years old (OR: 1.66; 95% CI: 1.22, 2.26), patients with ocular comorbidities (OR: 1.65; 95% CI: 1.53, 1.77), patients who had undergone surgery lasting more than 60 min (OR: 1.39; 95% CI: 1.14, 1.69), patients who had surgery without an intraocular lens (IOL) (OR: 1.64; 95% CI: 1.20, 2.26), and patients with postoperative complications (OR: 8.76; 95% CI: 8.13, 9.45). Worsening VA was significantly higher among male patients (OR: 1.11; 95% CI: 1.01, 1.22), patients who had ocular comorbidities (OR: 1.76; 95% CI: 1.59, 1.96), patients who had undergone surgery lasting more than 60 min (OR: 1.94; 95% CI: 1.57, 2.41), patients who had surgery without an IOL (OR: 2.03; 95% CI: 1.48, 2.80), and patients with postoperative complications (OR: 21.46; 95% CI: 19.35, 23.80). The factors impacting “no changes” in and “worsening” of VA after cataract surgery were the following: older age, male gender, ethnicity, ocular comorbidities, surgeon grade, absence of IOL, intraoperative complication, and postoperative problems.

## 1. Introduction

Blindness and visual impairment are part of the global burden of eye disease. In 2010, it was estimated that about 270.5 million people were living with visual impairment [1]. The leading cause of blindness among people 50 years and older was cataract, followed by glaucoma, under-corrected refractive error, age-related macular degeneration, and diabetic retinopathy [2]. 

The results of the National Eye Survey (NES) in 2014 revealed that the main causes of blindness in Malaysia were untreated cataracts, diabetic retinopathy, other posterior segment diseases, and glaucoma [3]. In the same survey, the prevalence of blindness was reported as 1.2%, severe visual impairment at 1.0%, and moderate visual impairment at 5.9%. Sabah and Sarawak (located on the Malaysian part of the island of Borneo) had the highest prevalence of blindness as well as moderate and severe visual impairment, possibly because these two states had the most inadequate access to ophthalmological services in the country [3]. 

The most effective way to treat cataract that causes visual impairment is cataract surgery. It is the world’s most commonly performed ocular procedure [4]. The standard technique for cataract surgery in Malaysia is phacoemulsification (phaco) [5]. Several international publications, including from Malaysia, have shown that the quality of vision before and after cataract surgery is associated with several factors [6,7,8,9,10,11]. For example, within the age group of 85 to 89 years old, 61.1% had poor vision after surgery, and age was a significant risk factor for having worse visual outcomes [7]. In addition, females had a higher risk of experiencing poor visual outcomes than males [10]. According to Lai et al. [6], poor visual outcomes after surgery were associated with older patients, the presence of systemic and ocular comorbidities, and complications during and after surgery. 

Most previous research focused on the factors associated with postoperative visual acuity (VA) following cataract surgery. Based on nationally representative data, this study aims to determine the factors affecting the improvement of VA before and after phaco surgery among cataract patients in Malaysia. 

## 2. Materials and Methods

This study was designed as a retrospective cohort study, where the patients’ out-comes and all clinical information from 2014 to 2018 were extracted from the National Eye Database (NED). The data sources for eye care providers in NED were mainly from the public hospitals and clinics. Data collection included demography, medical history, operative events, postoperative visual outcome, and potential causes for poor outcomes. The NED involved 72 data providers (SDP) all over Malaysia, who participated in data collection and data entry. The data were collected using case report forms (CRF) and were entered into the web application afterwards or directly into the web application during the clinical work. Authorized staff at each SDP were given passwords to perform data entry and manage the data [5]. All patients aged 19 years and older with primary causes of cataract who underwent phacoemulsification cataract surgery, and who were registered in the database between January 2014 until December 2018, were included in this study. 

The factors affecting VA improvement were divided into four main groups. The first group was “socio-demographic factors”, consisting of age, gender, race, and cause of cataracts. Age was defined as the age on the data collection date and was categorized into four main groups: ≤40, 41–60, 61–80, and >80. There are various races in Malaysia, which are Malay, Chinese, Indian, and others (including Orang Asli, Melanau, Kadazan/Murut/Bajau, Bidayuh, Iban, and foreigners). The causes of cataracts were recorded as either primary or secondary. This study only included primary cataract causes, which were further divided into senile/age-related, congenital, developmental, or others.

The second group was the “preoperative factor”, consisting of ocular and systemic comorbidities. The ocular comorbidities were pterygium, glaucoma, pseudoexfoliation, diabetic retinopathy, age-related macular degeneration (ARMD), and others. The ocular comorbidities analysis was measured as yes and no. The patients with any ocular comorbidities were considered positive (yes) ocular comorbidities. Systemic comorbidities were presented as yes and no. The patients with positive (yes) systemic comorbidities were when the patients had one or more types of systemic comorbidities, such as hypertension, diabetes mellitus, ischaemic heart disease, cerebrovascular accident, renal failure, COAD/asthma, and others.

The “surgical factor” formed the third group, where the information included the surgeon grade, duration of surgery, types of anaesthesia, and intraocular lens (IOL). Surgeons’ grades were further grouped into specialists, gazetting, and medical officers. The surgery duration was calculated from when surgery commenced to when it ended. It was further grouped into less than 30 min, 31 to 60 min, and more than 60 min. Even though several surgical techniques were used in Malaysia, such as phaco, lens aspiration, extracapsular cataract extraction (ECCE), and phaco converted to ECCE, only phacoemulsification surgery was included in this study. Types of anaesthesia were classified into the local and general groups, while for IOL, the analysis was done using IOL yes and IOL no.

The last group was the “complication factor”, where there were two complications to be studied: intraoperative and postoperative complications. The intraoperative complications included posterior capsule rupture, vitreous loss, drop nucleus, suprachoroidal haemorrhage, central corneal oedema, zonular dehiscence, and others. The analysis of intraoperative complications was presented as yes and no. The patients with one or more complications were considered as patients with intraoperative complications. Postoperative complications included high astigmatism, posterior capsular opacity, cystoid macular oedema, infective endophthalmitis, and others. Postoperative complications were measured as either yes or no, and if the patients had one or more postoperative complications, they were considered as patients with postoperative complications. In contrast, a patient without complications was regarded as having no complications.

The main outcome in this study was the improvement of VA and was categorized into three levels: worse, no change, and improved VA. The VA was measured twice: before surgery and at 12 weeks after surgery. The VA was recorded as 6/5, 6/6, 6/9, 6/12, 6/18, 6/24, 6/36, 6/60, 5/60, 4/60, 3/60, 2/60, 1/60, counting fingers (CF), hand movement (HM), perception light (PL), and no perception light (NPL), and converted into the Logarithm of the Minimum Angle of Resolution (LogMAR) value for analysis. The improvement in VA was calculated by subtracting the LogMAR value after and before surgery. A decrease of 0.1 in LogMAR means an improvement in visual acuity, while a LogMAR value increase of 0.1 indicates worsening visual acuity after surgery. Conversely, the LogMAR value of 0.0 indicated no changes in visual acuity before and after surgery [6].

The associations between demographic, preoperative, surgery, and complication factors with VA improvement were analysed using multinomial logistic regression analysis. All the factors were adjusted, and the adjusted odds ratio was expressed as adjusted odds ratio (Adj. OR) with a 95% confidence interval (CI).

## 3. Results

### 3.1. Descriptive Statistics

#### 3.1.1. Proportion of Improvement in Visual Acuity (VA) among Cataract Patients

A total of 180,776 cataract patients between 2014 and 2018 were included in the analysis. The proportion of vision improvement was 96.4% (95% CI: 96.3, 96.5), followed by patients with no change in vision after surgery 2.3% (95% CI: 1.8, 2.8) and worsening vision 1.3% (95% CI: 0.8, 1.8) (Table 1).

#### 3.1.2. Socio-Demographic Factors

Patients’ demographic characteristics are shown in Table 2. The patients’ ages ranged from 19 to 99 years old, with a mean of 66.7 years and a standard deviation of 8.96. The majority of adult patients in this study were 61–80 years old (73.1%), 21.6% were between 41–60 years old, and 4.6% were more than 80 years. A higher proportion of females was observed (53.5% vs. 46.5%). Both genders showed a higher proportion of improving VA than no change in and worsening of VA (Table 2).

The ethnic distribution was 41.5% Malay, 31.9% Chinese, and 12.4% Indian, while other races included Orang Asli, Sabahan, Sarawakian, and foreigners (6.8%). Most of the causes were primary; senile or age-related was the highest (95.6%), and the developmental cause of cataract was only 0.3%. After undergoing surgery, all the causes of cataracts get improved the visual acuity (Table 2).

#### 3.1.3. Comorbidities and Complication Factors

Out of all patients, 33.6% had ocular comorbidities in which patients with no change and worsening vision had 4.0% and 2.6% ocular comorbidities, respectively. Meanwhile, patients with improved vision had 93.4% ocular comorbidities. Most patients with cataract had systematic comorbidities (80.0%). Patients with worsening vision improvement were 1.3% and no change in vision were 2.4% among the cataract patients with systemic comorbidities. Meanwhile, 96.3% of cataract patients with systemic comorbidities had improved vision after surgery (Table 3).

Table 3 shows that most patients did not develop complications during cataract surgery (97.1%). Overall, the proportion of cases having intraoperative complications was small (2.9%). From this, 3.5% of the patients had worsening vision, while 3.9% had no change in vision after surgery. For the postoperative complications factors, 92.3% of patients did not have postoperative complications, and most patients had improved vision after surgery (98.1%). The proportion of patients with worsening VA who had postoperative complications was 10.9%, and 12.6% of patients had no changes in vision after surgery.

#### 3.1.4. Surgical Factors

The surgical factors of cataract patients are described in Table 4. Most surgeries were performed by specialists (90.5%), 5.1% by gazetting specialists, and 4.1% by medical officers. Out of all patients, 77.0% had undergone less than 30 min of cataract surgery. Among patients with a duration of surgery of less than 30 min, 96.7% had improved VA, only 2.2 % had no change in vision, and 1.1% had worsening VA. Most patients had an IOL implant or lens replacement (99.6%). Those patients with no IOL implant had worsening vision after cataract surgery (8.5%) compared to patients with an IOL implant. A total of 95.0% of patients underwent surgery under local anaesthesia. Among those patients who underwent surgery under general anaesthesia, 2.2% experienced worsening VA, and 2.7% had no changes in VA after a 12-week follow-up (Table 4).

### 3.2. Multivariable Analysis

#### 3.2.1. Detailed Analysis Comparing No Changes in VA and Improved VA

**Figure 1:**
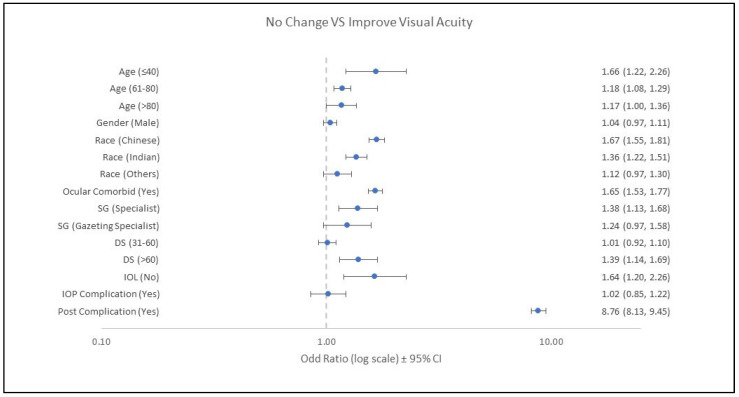
Forest plot of no change VA versus improved VA (ref). Reference for age was 41–60 years old, gender was female, race was Malay, ocular comorbid was no, surgeon grade (SG) was medical officer, duration of surgery (DS) was ≤30 min, intraocular lens (IOL) was yes, intraoperative complication (IOP) was no, and postoperative complication (Post) was no.

Multinomial logistic regression analysis was conducted to determine the factors associated with VA improvement after undergoing surgery. In the logistic regression model, a factor with a confidence interval that does not include one (1) indicates that it is significantly associated with the risk of having no change in VA after undergoing cataract surgery. The analysis results are illustrated in Figure 1. The figure shows that those aged less than 40 years and 61–80 years were at higher risk of having no change in VA, with an OR of 1.66 (95% CI: 1.22, 2.26) and 1.18 (95% CI: 1.08, 1.29), respectively. The distribution of male and female patients was equal in no change in VA (Table 2), but male patients had a 1.04 times (95% CI: 0.97, 1.11) higher risk of no change in VA compared to female patients. Chinese and Indians patients were at greater risk of having no change in VA, while Malay patients had a better chance of improved VA. Ocular comorbidities were significantly associated with a higher chance of postoperative visual no change (OR: 1.65; 95% CI: 1.53, 1.77). Surgeon grades were significantly associated with the outcome (*p* = 0.001). Surgery by a specialist has 1.38 times more risk of no change in vision after surgery (95% CI: 1.13, 1.68). The duration of surgery is one of the important factors in the improvement of VA after surgery. In this study, the duration of surgery of more than 60 min was significantly associated with a higher chance of having the no change VA than surgery of less than 30 min (OR: 1.39; 95% CI: 1.14, 2.26). An IOL was found to significantly affect visual improvement. Patients without an IOL implant had 1.64 times (95% CI: 1.20, 2.26) higher risk of no change in VA after surgery than patients with an IOL implant. Patients with intraoperative complication (OR: 1.02; 95% CI: 0.85, 1.22) and postoperative complications (OR: 8.76; 95% CI: 8.13, 9.46) had a significantly higher chance of no change in visual acuity after cataract surgery. The results are demonstrated in Figure 1.

#### 3.2.2. Detail Analysis Comparing Worsening VA and Improved VA

**Figure 2:**
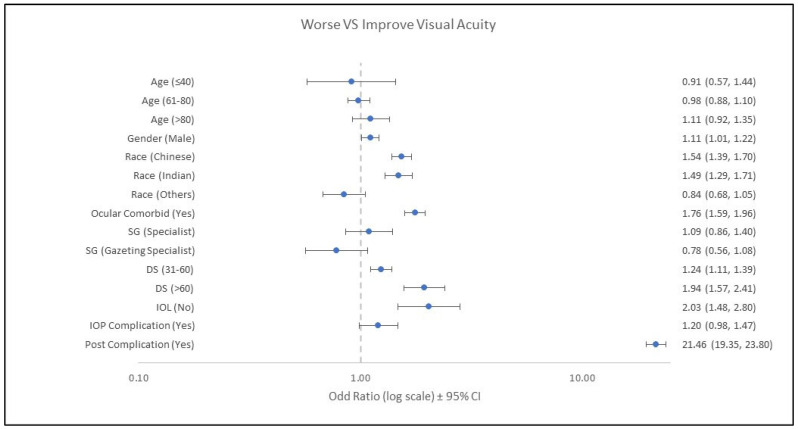
Forest plot of worsening VA versus improved VA (ref). Reference for age was 41–60 years old, gender was female, race was Malay, ocular comorbid was no, surgeon grade (SG) was medical officer, duration of surgery (DS) was ≤30 min, intraocular lens (IOL) was yes, intraoperative complication (IOP) was no, and postoperative complication (Post) was no.

The analysis results are shown in Figure 2. In the logistic regression model, a factor with a confidence interval that does not include one (1) indicates that it is significantly associated with the risk of having worsening VA after undergoing cataract surgery. Those over 80 years were at higher risk of worsening VA, with an OR of 1.11 (95% CI: 0.92, 1.35) compared to the other age groups. The worsening VA distribution between males and females was similar (Table 2) but males were 1.11 times (95% CI: 1.01, 1.22) more likely to get risk of worsening VA compared to females. Chinese and Indian patients were at greater risk of worsening VA, while Malay patients had a better chance of improving VA. Ocular comorbidities were significantly associated with a higher chance of postoperative visual worsening (OR: 1.76; 95% CI: 1.59, 1.96). An IOL can significantly affect visual improvement. Duration of surgery was significantly associated with the outcome (*p* < 0.001). Surgeries that take 31 to 60 min have a risk of 1.24 (95% CI: 1.11, 1.39) times of having worsening VA, and a period of surgery that exceeds 60 min will have a 1.94 (95% CI: 1.57, 2.41) times higher risk of getting worse vision compared to a surgical period of less than 30 min. Patients without an IOL implant had 2.03 (95% CI: 1.48, 2.80) times higher risk of worsening in VA compared to patients with an IOL implant. Both intraoperative and postoperative complications were associated with a greater risk of a worsening VA (OR: 1.20; 95% CI: 0.98, 1.47 and OR: 21.46; 95% CI: 19.35, 23.80, respectively) (Figure 2).

## 4. Discussion

This paper showcases a study of the clinical outcomes of cataract surgery among adults (≥19 years old) in Malaysia involving 43 hospitals and 29 cataract outreach locations under the Malaysian Ministry of Health between 2014 and 2018. Of all adult patients, 96.4% had improved visual acuity (VA) after surgery. The proportion of patients who experienced no change in VA after surgery was 2.3%, and the proportion who experienced worsening VA was 1.3%. In previous studies, the average age for cataract patients was between 64.5 and 75.0 years old [7,9,11,12]; similarly, in this study, 73.1% were between 61 and 80 years old. Age is a significant factor affecting visual acuity improvement after undergoing cataract surgery. According to Lai et al. [6], older patients with a lower chance of experiencing visual improvement following surgery were patients with complications during surgery and those with comorbidities [6]. Another study also indicated that patients aged 80 years and up had a significantly higher risk of experiencing impaired and poor visual outcomes following surgery [9]. However, the findings from the logistic regression analysis of our study showed that patients under 40 years old had a higher risk of experiencing no change in VA (OR: 1.66; 95% CI: 1.22, 2.26) when compared to patients aged 41 to 60 years old, after adjusting for comorbidities, surgery factors, and complications. Meanwhile, patients older than 61 were also at risk of experiencing no change and worsening visual acuity. This may be related to the presence of ocular complications and comorbidities. This could be seen in the logistic regression analysis, where there was a significant relationship between patients with complications or comorbidities and an improvement in VA. 

In this study, the proportion of females with cataracts (53.5%) is slightly higher than males (46.5%) during the 2014–2018 timeframe. Similarly, a systematic review of global causes of blindness and distance vision impairment from 1990–2020 showed that females with cataracts were more common than males with cataracts [13]. According to Khanna et al. [11], gender was related to the visual outcomes, and females have a lower likelihood of experiencing improved visual outcomes than males. In our study, gender is a significant factor related to visual improvement. However, by using logistic regression, we see that males are 4% more likely to have no change in VA following cataract surgery and 11% more likely to have worsening VA than females. Malaysia is known for having many ethnicities; most of the cataract patients were Malay (41.5%), followed by Chinese (31.9%), Indian (12.4%), and other races (6.8%). This is roughly representative of the ethnic distribution of the population in Malaysia. When comparing visual improvement after cataract surgery across ethnicities, Chinese, Indian, and other races were more likely to experience no change in VA and worsening VA than Malays. These results differ from a study published by Thevi and Godinho [9], who reported that Malay patients had an increased risk of experiencing poor visual outcomes, while Chinese and Indian patients had a better chance of a good visual outcome. 

A Malaysian study by Thevi et al. [8] reported that 98% of cataract causes were primary, and this included senile, developmental, and congenital cataracts. The cause of cataracts was not statistically associated with intraoperative complications and visual improvement after cataract surgery. Similarly, in studies in Nigeria, cataract causes did not significantly relate to good VA following surgery. However, the senile group had the highest number of good visual outcomes following surgery [14]. A recent study reported that patients with age-related cataracts and who had intraoperative complications were not significantly associated with poor visual outcomes, but those with age-related cataracts and ocular comorbidities had significant poor visual outcomes [11]. The current study’s findings agree with previous research. The causes of cataracts were not associated with visual improvement (*p* = 0.132). In our study, 95.6% of the patients had senile or age-related cataracts, and among those patients, 96.4% experienced improved VA, 1.3% experienced worsening vision, and 2.3% did not experience any vision changes after surgery.

Among the patients in this study with systemic comorbidities, hypertension was the most prevalent proportionally, followed by diabetes mellitus and ischaemic heart disease. However, the logistic regression analysis showed that systemic comorbidities did not affect the worsening of or improvement in VA regardless of adjustment with or without other variables such as age, gender, surgery factors, and intraoperative and postoperative complications. Our study’s findings are consistent with those of Lai et al. [6], who reported hypertension, diabetes mellitus, myocardial infarction, and cerebrovascular disease as the most common systemic comorbidities. In addition, the systemic comorbidities were not significantly associated with visual improvement (*p* = 0.33) [6]. Other studies also reported that systemic comorbidities were not associated with intraoperative and postoperative complications. At the same time, systemic comorbidities also did not affect visual improvement following cataract surgery [8].

Ocular comorbidities are always related to a vision-related disability, including cataracts [15]. A study from Australia showed that in patients over 60 years old who underwent cataract surgery, most have age-related macular degeneration (ARMD), glaucoma, or diabetic retinopathy [16]. Very old patients (over 80 years old) with ocular comorbidities are at higher risk of experiencing poor postoperative VA, with the most common comorbidities being ARMD and diabetic retinopathy [17]. Our study showed that the most common ocular comorbidity was diabetic retinopathy, followed by glaucoma and ARMD. However, from the logistics regression analysis results, patients with any ocular comorbidity had a high risk of experiencing no change in VA and worsening VA after surgery (OR: 1.65, 95% CI: 1.53, 1.77, and OR: 1.76, 95% CI: 1.59, 1.96, respectively).

This study showed that most surgeries were performed by specialists (90.5%). Currently, phaco is most surgeons’ preferred method for cataract surgery. This is because the wound is small and heals rapidly [4]. The majority of studies report that the procedure to treat cataracts uses phaco and extracapsular cataract extraction (ECCE) [18,19,20,21,22,23]. According to Abdulsalam [22], phaco achieves good postoperative VA compared to ECCE techniques. Based on his study, 88.9% of eyes operated on using the phaco technique achieved a VA of 6/18 or better, while only 68.5% of eyes operated on using the ECCE technique achieved a VA of 6/18 or better, and this difference was statistically significant [22]. Only patients undergoing cataract surgery using the phaco method were selected in our study. When comparing specialists and medical officers who perform surgery, patients operated on by specialists have 1.38 (95% CI: 1.13, 1.68) times the risk of experiencing no change in visual acuity. This is because specialists will perform the more complicated surgeries, and the chances of getting good results in these surgeries are lower. Complicated surgery is caused by a patient’s eye condition, including patients with diabetic retinopathy or glaucoma [9].

An IOL is an artificial lens used to replace the cloudy lens in the eyes of patients with cataracts [24]. In this study, about 99.6% of the cataract surgeries were done with IOL implantation, and most patients had visual improvement. The result from the logistic regression analysis showed that IOLs had a significant association with visual improvement. Patients who had undergone surgery without IOLs had 1.6 times the risk of experiencing no change and 2.0 times the risk of experiencing worsening VA after adjusting for other factors. This finding agrees with Matta et al. [10], which showed that eyes without IOLs or anterior chamber intraocular lenses (AC-IOLs) are 12.6 (95% CI: 2.65, 60.25) times more likely to have poor visual outcomes. As shown in other studies, eyes without IOLs are one of the risk factors for poor visual outcomes [9,22,25]. 

Most of the surgeries were performed in under 30 min (77%), followed by 31 to 60 min (16.5%), and in some cases, surgery lasted more than 60 min (1.8%). Surgery duration was a significant factor affecting the improvement of VA. Patients who had undergone surgery lasting between 31 and 60 min had 1.24 times the risk of experiencing worsening VA after surgery compared to surgery lasting less than 30 min. Patients who had surgery that lasted more than 60 min had 1.94 times the risk of experiencing worsening VA, and 39% of the time did not experience any vision changes after surgery compared to patients who had undergone surgery under 30 min. According to Thanigasalam et al. [8], the duration of cataract surgery significantly correlates with surgery type. A surgery duration shorter than 30 min is standard for phaco procedures. For patients who underwent ECCE and “phacoemulsification converted to ECCE” procedures, it took approximately 31 to 60 min to complete the operation [8]. The most recent study on surgical duration showed that the shorter surgeries of approximately 30 min or less 30 min were 2.1 (95% CI: 1.95, 2.32) times more likely to be associated with good visual outcomes. Meanwhile, surgeries that took more than 60 min were 3.3 (95% CI: 1.72, 3.96) times more likely to have poor visual outcomes [9]. 

In our study, the most common complications were posterior capsular rupture, vitreous loss, and zonular dehiscence. A previous study by Konstantopoulos et al. [26] reported that vitreous loss was significantly associated with poor postoperative VA [26]. In general, pre- and post-surgery VA is significantly different between patients with and without intraoperative complications [27]. Our study had the same findings as the previous studies: intraoperative complications related to the visual outcome after surgery. There were 1.02 (95% CI: 0.85, 1.22) times the risk of experiencing no change in VA and 1.20 (95% CI: 0.98, 1.47) times the risk of experiencing worsening VA after surgery for patients with intraoperative complications compared to those without intraoperative complications.

Cataract surgery is safe in more than 95% of patients [28]. The percentage of those who experience complications is small, but these complications can affect the visual outcome. A report from Chan et al. [28] shows that posterior capsular opacification (PCO) is the most common complication after cataract surgery [28]. A study from Taiwan supports the study from Chan et al. (2010), showing that 57% of the study sample developed PCO after cataract extraction [29]. There is a different finding in the study by Lundström et al. (2013), where the most frequent postoperative complications were endophthalmitis and central corneal oedema. Those complications also have the highest frequency of worse visual outcomes. [7]. However, our study found that the most frequent postoperative complication is high astigmatism, followed by cystoid macular oedema and posterior capsular opacity, and that the type of complications is slightly different from those in the studies by Chan et al. (2010) and Lundström et al. (2013). The result of the logistic regression analysis showed postoperative complications as a significant factor affecting the improvement of visual outcomes. Patients with any postoperative complications had 8.76 (95% CI: 8.13, 9.45) times more risk of experiencing no change in VA and a 21.46 (95% CI: 19.35, 23.80) times higher risk of experiencing worsening VA after surgery.

The limitation of this study is the study design (cohort retrospective), which is susceptible to systematic bias. Clinical factors affecting the improved VA among patients who have undergone cataract surgery can be obtained through prospective population-based studies with a large sample size. For such a survey it is best to include contrast sensitivity and the patient’s quality of life (patient-reported outcome measurement (PROM)).

## 5. Conclusions

Overall, the outcomes of cataract surgery in Malaysia are good, and most patients experience improved VA after surgery. However, there are also situations where patients experienced no changes in or worsening VA after surgery. Factors contributing to worsening VA are increasing age, male gender, being non-Malay, the presence of ocular comorbidities, the surgeon’s ability, the surgery’s duration, the absence of the intraocular lens, and the presence of intraoperative and postoperative complications. These findings provide evidence-based information for relevant clinicians and policymakers in planning and implementing interventional programs.

A recommendation to improve this study is to take other “quality of life” measures, such as daily activities before and after surgery, socioeconomic factors, etc., which would be more interesting to study instead of just comparing VA before and after surgery. Some studies abroad measured the health development of cataract patients beyond just VA [30,31]. Additionally, there needs to be an improvement in the data collection in the National Eye Database (NED). Other factors can influence the improvement in visual acuity for cataract patients, such as their level of education and economic status [32]. From the education level and economic status data, we can understand the level of knowledge about eye care and how it is practiced among cataract patients and the financial burden of obtaining cataract treatment.

## Figures and Tables

**Table 1:** Proportion of improvement in VA among cataract patients in Malaysia.

Visual Acuity	n	%	95% CI
Improve	174,256	96.4	(96.3, 96.5)
No change	4223	2.3	(1.8, 2.8)
Worsening	2297	1.3	(0.8, 1.8)

**Table 2:** Socio-demographic factors according to the level of improved VA.

Variables	Visual Improvement				
	Total	Worse	No Change	Improve	*p*-Value ^a^
	180,776	(n = 2297)	(n = 4223)	(n = 174,256)	
		n (%)	n (%)	n (%)	
Age (Years)					
≤40	1257 (0.7)	29 (2.3)	56 (4.5)	1172 (93.2)	<0.001
41–60	39,023 (21.6)	513 (1.3)	801 (2.1)	37,709 (96.6)	
61–80	132,196 (73.1)	1575 (1.2)	3090 (2.3)	127,531 (96.5)	
>80	8300 (4.6)	180 (2.2)	276 (3.3)	7844 (94.5)	
Gender					
Female	96,647 (53.5)	1167 (1.2)	2180 (2.3)	93,300 (96.5)	0.002
Male	84,129 (46.5)	1130 (1.3)	2043 (2.4)	80,956 (96.2)	
Races					
Malay	75,045 (41.5)	821 (1.1)	1411 (1.9)	72,813 (97.0)	<0.001
Chinese	57,621 (31.9)	906 (1.6)	1741 (3.0)	54,974 (95.4)	
Indian	22,324 (12.4)	307 (1.4)	513 (2.3)	21,504 (96.3)	
Others	12,292 (6.8)	98 (0.8)	241 (2.0)	11,953 (97.2)	
Missing	13,494 (7.5)	165 (1.2)	317 (2.3)	13,012 (96.4)	
Cause of Cataract					
Senile/Age-Related	172,736 (95.6)	2217 (1.3)	4055 (2.3)	166,464 (96.4)	0.132 ^b^
Development	384 (0.2)	5 (1.3)	8 (2.1)	371 (96.6)	
Others	94 (0.1)	3 (3.2)	5 (5.3)	86 (91.5)	
Missing	7562 (4.2)	72 (1.0)	155 (2.0)	7335 (97.0)	

^a^ Chi-square test. ^b^ Fisher’s exact test.

**Table 3:** Comorbidities and complication factors according to the level of improved visual acuity.

Variables	Visual Improvement				
	Total	Worsening	No Change	Improve	*p*-Value ^a^
	180,776	(n = 2297)	(n = 4223)	(n = 174,256)	
		n (%)	n (%)	n (%)	
Ocular Comorbidities					
No	120,045 (66.4)	732 (0.6)	1793 (1.5)	117,520 (97.9)	<0.001
Yes	60,731 (33.6)	1565 (2.6)	2430 (4.0)	56,736 (93.4)	
Systemic Comorbidities					
No	36,189 (20.0)	416 (1.1)	808 (2.2)	34,965 (96.6)	0.023
Yes	144,587 (80.0)	1881 (1.3)	3415 (2.4)	139,291 (96.3)	
Intraoperative Complication					
No	175,605 (97.1)	2118 (1.2)	4020 (2.3)	169,467 (96.5)	<0.001
Yes	5171 (2.9)	179 (3.5)	203 (3.9)	4789 (92.6)	
Postoperative Complication					
No	166,866 (92.3)	787 (0.5)	2464 (1.5)	163,615 (98.1)	<0.001
Yes	13,910 (7.7)	1510 (10.9)	1759 (12.6)	10,641 (76.5)	

^a^ Chi-square test.

**Table 4:** Surgical factors according to the level of improved VA.

Variables	Visual Improvement				
	Total	Worsening	No Change	Improve	*p*-Value ^a^
	180,776	(n = 2297)	(n = 4223)	(n = 174,256)	
		n (%)	n (%)	n (%)	
Surgeon Status					
Specialist	163,525 (90.5)	2117 (1.3)	3896 (2.4)	157,512 (96.3)	<0.001
Gazetting Specialist	9292 (5.1)	93 (1.0)	194 (2.1)	9005 (96.9)	
Medical Officer	7437 (4.1)	85 (1.1)	129 (1.7)	7223 (97.1)	
Missing	522 (0.3)	2 (0.4)	4 (0.8)	516 (98.9)	
Duration of surgery (minutes)					
≤30	139,263 (77.0)	1491 (1.1)	3052 (2.2)	134,720 (96.7)	<0.001
31–60	29,854 (16.5)	541 (1.8)	785 (2.6)	28,528 (95.6)	
>60	3250 (1.8)	142 (4.4)	156 (4.8)	2952 (90.8)	
Missing	8409 (4.7)	123 (1.5)	230 (2.7)	8056 (95.8)	
Intraocular Lens (IOL)					
Yes	180,019 (99.6)	2234 (1.2)	4165 (2.3)	173,620 (96.4)	<0.001
No	737 (0.4)	63 (8.5)	57 (7.7)	617 (83.7)	
Missing	20 (0.0)	0 (0.0)	1 (5.0)	19 (95.0)	
Anaesthesia in Cataract Surgery					
Local	171,662 (95.0)	2119 (1.2)	3984 (2.3)	165,559 (96.4)	<0.001
General	6760 (3.7)	147 (2.2)	184 (2.7)	6429 (95.1)	
Missing	2354 (1.3)	31 (1.3)	55 (2.3)	2268 (96.3)	

^a^ Chi-square test.

## Data Availability

The data presented in this study are available on request from the corresponding author. The data are not publicly available due to ethical restrictions.
